# Neural Correlates of Behavioural Olfactory Sensitivity Changes Seasonally in European Starlings

**DOI:** 10.1371/journal.pone.0014337

**Published:** 2010-12-15

**Authors:** Geert De Groof, Helga Gwinner, Silke Steiger, Bart Kempenaers, Annemie Van der Linden

**Affiliations:** 1 Bio-Imaging Lab, University of Antwerp, Antwerp, Belgium; 2 Department of Behavioural Ecology and Evolutionary Genetics, Max-Planck Institute for Ornithology, Starnberg, Germany; Harvard University, United States of America

## Abstract

**Background:**

Possibly due to the small size of the olfactory bulb (OB) as compared to rodents, it was generally believed that songbirds lack a well-developed sense of smell. This belief was recently revised by several studies showing that various bird species, including passerines, use olfaction in many respects of life. During courtship and nest building, male European starlings (*Sturnus vulgaris*) incorporate aromatic herbs that are rich in volatile compounds (e.g., milfoil, *Achillea millefolium*) into the nests and they use olfactory cues to identify these plants. Interestingly, European starlings show seasonal differences in their ability to respond to odour cues: odour sensitivity peaks during nest-building in the spring, but is almost non-existent during the non-breeding season.

**Methodology/Principal Findings:**

This study used repeated *in vivo* Manganese-enhanced MRI to quantify for the first time possible seasonal changes in the anatomy and activity of the OB in starling brains. We demonstrated that the OB of the starling exhibits a functional seasonal plasticity of certain plant odour specificity and that the OB is only able to detect milfoil odour during the breeding season. Volumetric analysis showed that this seasonal change in activity is not linked to a change in OB volume. By subsequently experimentally elevating testosterone (T) in half of the males during the non-breeding season we showed that the OB volume was increased compared to controls.

**Conclusions/Significance:**

By investigating the neural substrate of seasonal olfactory sensitivity changes we show that the starlings' OB loses its ability during the non-breeding season to detect a natural odour of a plant preferred as green nest material by male starlings. We found that testosterone, applied during the non-breeding season, does not restore the discriminatory ability of the OB but has an influence on its size.

## Introduction

It has long been thought that birds lack a well-developed sense of smell. However during the past 20-30 years many studies have shown the complexity of avian olfactory structures, not only in species with larger olfactory bulbs (OB) like vultures, petrels and albatrosses but also in species with much smaller OB such as pigeons, quail, robins, hummingbirds and starlings (for review see [1], [2], [3]). Birds use their sense of smell in navigation, in avoidance of insects, in nest identification, in detection of chemical signals during courtship, in food searching and in avoiding predators [4].

Male European starlings (*Sturnus vulgaris*) carry fresh green plants into their nest holes. Starlings prefer to incorporate plants rich in volatile compounds like milfoil (*Achillea millefolium*) over other green plant species [5]. Because parasite and pathogen load increase with repeated nest use [6], aromatic herbs serve as a fumigant protecting nestlings [7]. Interestingly, European starlings can only discriminate plant odours and use their sense of smell to identify odorous nest material at the time of the year when courtship and nest building takes place [8]. The underlying structural changes that may occur in these seasonal shifts in olfactory acuity are however still unknown. We hypothesized that the volume of the OB may increase with the reproductive season similar to the volume changes of the song nuclei of songbirds [9]. In starlings, plasma testosterone levels increase towards the breeding season and this increase is associated with courtship and nest building behaviour [10]. Testosterone may therefore be a candidate to stimulate the olfactory system. However, the possible relation between olfactory acuity and steroids is not yet known.

Although being completely different species, both humans and starlings show many similarities in olfactory anatomy and functionality. Both species are considered to be microsmic, but both have shown to have a sense of smell that is more important than generally realized [8], [11]. Moreover, just as humans, birds lack a (functional) vomeronasal organ [12], [13] and the number of functional olfactory receptor genes is quite similar between songbirds and humans (between 200 and 350) [14], [15].

Manganese-enhanced MRI (MEMRI) is an *in vivo* method to map neuronal function and to trace neuronal connections in the olfactory and visual pathways of rodents [16]–[19] and the song control system of songbirds [20]–[22]. Here, we repeatedly imaged by MEMRI the brain of fourteen individual male starlings during the breeding and the non-breeding season. In this way we were able to assess the olfactory detection for milfoil and quantify between-season variation in the volume of the OB. We also studied the effect of testosterone (T) (by using T implants) on the OB.

## Materials and Methods

### Subjects

14 hand raised, adult male European starlings (*Sturnus vulgaris*; ±75 g) from a starling colony in Upper Bavaria, South Germany, were maintained in large outdoor aviaries at the Max Planck Institute for Ornithology (Seewiesen, Germany). During the experiments at the University of Antwerp (Belgium) they were housed in two indoor cages (1.40×2.20×2.10 m) under an artificial light-dark cycle simulating the natural photoperiod. Food and water were available ad libitum. During the breeding period (April/May) 3 nest boxes and 3 females per aviary were available as an additional reproductive stimulus [23]. All birds were individually marked with a numbered metal ring and colour bands.

### Ethics statement

Permission to take birds from the nests for scientific purposes was given by the Regierung von Oberbayern (reference number 820-8642.3-5/02). All experimental procedures were approved by the Committee on Animal Care and Use at the University of Antwerp, Belgium.

### Experimental setup

The experiments were conducted once during the breeding season (Spring: 16 April - 8 May 2007), and were repeated during the non-breeding season (Summer: 13 August - 9 September 2007), when birds have become photorefractory, their gonads are fully regressed, and testosterone levels are low. In each experiment the birds were measured twice, once with and once without milfoil as olfactory stimulus with a 2 week period in between measurements. The order of these two stimuli conditions was randomized between birds.

To test if testosterone is directly responsible for potential effects between the breeding and non-breeding season, we implanted eight of the 14 birds with testosterone implants in the neck region after the second MRI experiment, while the others received empty implants. 12-mm long capsules of Silastic tubing (Dow Corning, Midland, MI) were filled with crystalline testosterone and sealed on both ends with liquid silicone. The implants caused no infections and wounds healed entirely within 5 days. However, three starlings lost their T-implant after 2 to 4 days and were added to the empty implanted group. Three weeks after implantation, the MRI experiments were performed again (Fall: 5 November - 23 November 2007).

### Manganese enhanced MRI protocol

Birds were anaesthetized with an initial intramuscular (chest) injection of 5 ml/kg of a mixture containing 0.33 ml xylazine (Rompun: 20 mg/ml), 2.10 ml ketamine (Ketalar: 50 mg/ml) and 4.33 ml saline solution. They were then kept anaesthetized during the whole experiment with the anaesthesia mixture at a rate of 0.15 ml/h through a chest catheter (Micro-Flo, 27GA, DKS Overscan, Milano, Italy). Body temperature was continuously monitored (SA instruments, Stony Brook, NY) and automatically regulated (with a warm air feedback system, SA instruments) within a narrow range of 40–41°C. After anaesthesia induction, the birds received a 10 µl injection in each nostril of a 40 mM Manganese Chloride (MnCl_2_) solution. Immediately after MnCl_2_ injection birds were exposed to an olfactory stimulus by placing 10 cm before the bird's beak a paper towel with two drops of milfoil oil, *Achillea millefolium*, (Neumond, natural fragrance (5 ml)) and this during 15 minutes in a periodic fashion (5 min direct exposure, 5 min no direct exposure (paper towel put away in an enclosed box), 5 min direct exposure). We chose milfoil because it is highly preferred as green nest material by the starlings from our study population [5].

Manganese is a MRI contrast agent that shortens the T_1_ time of protons. This tissue-specific (different for different biological tissues) time constant for protons, is a measure of the time taken to realign with the external magnetic field. The T_1_ constant will indicate how quickly the spinning nuclei will emit their absorbed RF into the surrounding tissue. By shortening this time period the tissues will appear brighter on T_1_-weighted MRI images.

Uptake of manganese into living cells is activity dependent: manganese is a calcium analogue that enters neurons via voltage-gated Ca^2+^ channels [24]. These Ca^2+^ channels open due to specific stimuli, in this case an odour. Once in cells, manganese is packaged into vesicles and transported in an anterograde direction along neurons [24]. Changes in the accumulation of manganese in target nuclei reflect in a fairly direct manner the activity of the neurons where manganese was introduced and taken up. This technique was indeed used previously to visualize changes in the activity of the olfactory pathway in mice [19].

**Figure 1:**
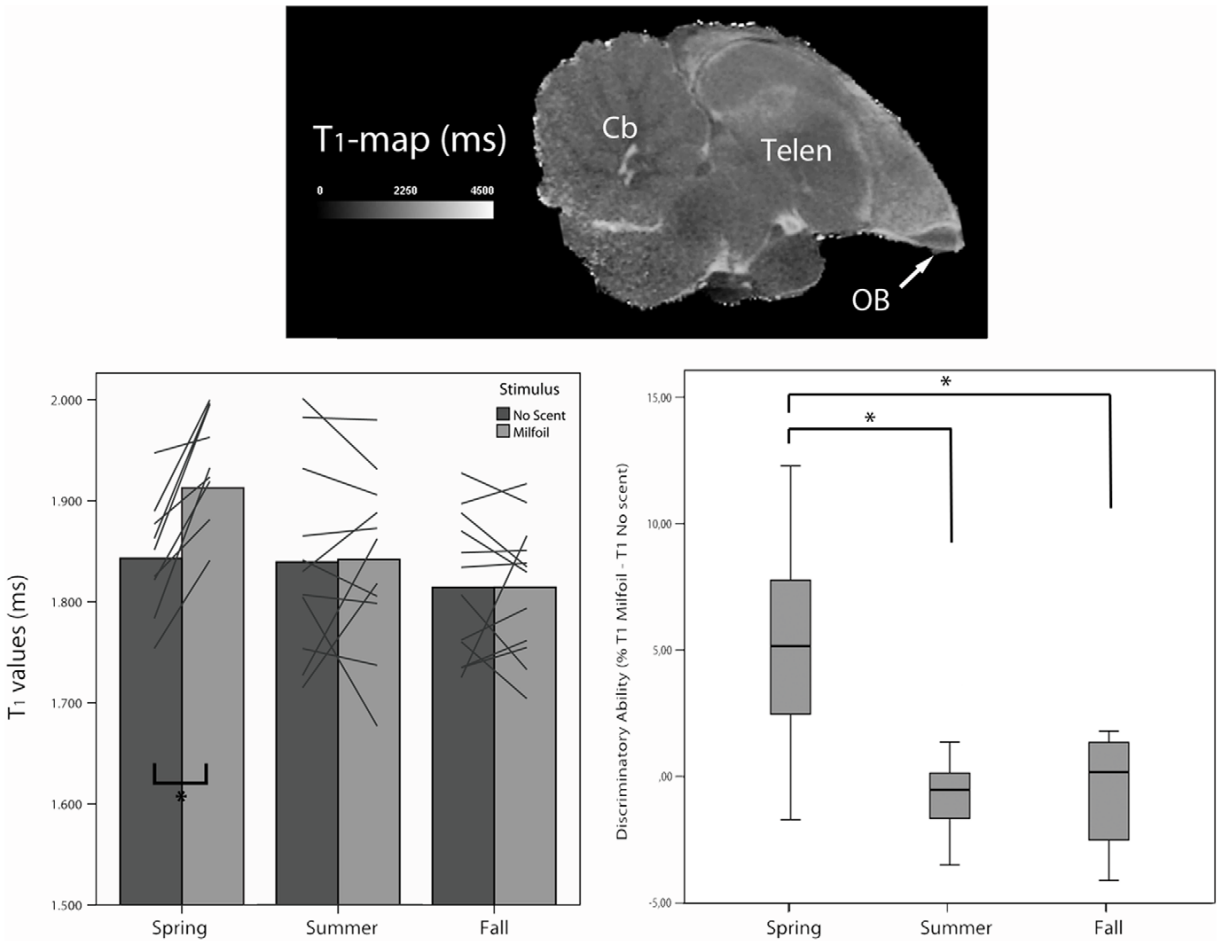
T_1_ values and ‘discriminatory ability’ of the olfactory bulb (OB) in different seasons. **Top**: Sagittal T_1_-map of one starling stimulated with milfoil during the breeding season. Notice the small size of the olfactory bulb (OB) compared to the rest of the brain and the accumulation of manganese chloride in the bulb resulting in a darker contrast (shorter T_1_) on T_1_-maps. Cb  =  Cerebellum; Telen  =  Telencephalon. **Bottom left**: T1 values of the olfactory bulb of the same individuals (N = 14) at different times of the year. In each season each animal was measured twice, once with a milfoil scent as stimulus, once without any scent. Lines represent measurements per starling per season. (*p<0.05). **Bottom right**: Boxplots of discriminatory ability [100×(T1 milfoil - T1 no scent)/T1 no scent)] of the same individuals (N = 14) at different times of the year. Error bars represent standard deviation. (*p<0.05).

Imaging was performed on a horizontal MR system (Biospec 94/20 USR, Bruker Biospin, Germany) with a magnetic field strength of 9.4T and the standard Bruker cross coil setup being a quadrature transmit coil and a rat head quadrature receive surface coil. A T1-weighted SE 3D RARE was obtained 40 min after MnCl2 injection. This 3D consisted of a field of view of 25 mm^3^ covering the whole starling brain. Additional image parameters were: spectral width = 50 kHz, TE = 7.5 ms, TR = 386 ms, RARE factor = 4, averages = 1 and acquisition matrix  = 256×128×128 ending up with a resolution of 0.098×0.195×0.195 mm^3^. This 3D sequence took approximately 20 minutes. Immediately following this 3D multiple 2D Inversion Recovery (IR) SE sequences were acquired consisting of one sagittal slice covering the OB. Each sequence had the following parameters: field of view  = 25 mm^2^, spectral width = 50 kHz, TE = 7.5 ms, TR = 12 s, RARE factor = 8, averages = 1, slice thickness = 0.5 mm and acquisition matrix  = 256×256 ending up with an in-plane resolution of 0.098×0.098 mm^2^. This sequence was repeated 10 times changing only the inversion time (TI = 70, 300, 750, 800, 900, 1100, 1250, 2500, 3000, 11000 ms) of which T1-maps could be calculated (**Fig. 1**). The ten sequences took approximately 50 min to obtain. All starlings recovered perfectly after each MR experiment.

### Beak colour

The beak colour of all subjects was assessed during fall. Beak colour in European starlings is dependent on plasma T [25], [26], changing from black in summer (when plasma T levels are basal) to yellow in spring (when plasma T levels are higher). It was recorded on an arbitrary scale of 0 (bill entirely black, from base to tip) to 5 (bill entirely yellow) [27].

### Testosterone Assay

After the MRI of fall, blood samples were taken from each male to assay T concentrations. The alar wing vein was punctured and 300–500 µl of blood was collected into heparinized tubes. The blood was transferred into centrifuge tubes and centrifuged at 7000 rpm for 15 min. The plasma was removed and stored in vials at −70°C until assayed for T. Frozen plasma samples were sent to the endocrine laboratory of the Leibniz Institute for Zoo and Wildlife Research in Berlin, Germany, where T levels were determined by enzyme immunoassays. The assay used a polyclonal antibody raised in rabbits against testosterone-11-hemisuccinate-BSA, and the label was testosterone-3-carboxymethyl-oxime-horse radish peroxidase. The testosterone standard curve ranged from 0.4 pg per 20 µl to 50 pg per 20 µl, and the cross-reactivity with testosterone was 100%, with 5α-dihydrotestosterone 10%, with androstenedione 2%, with estradiol <0.1%, and with progesterone <0.1%. The results are given in nanograms per millilitre of serum. The intra- and interassay coefficients of variation (CVs) were 8.9% and 12.3%, respectively (for details on the methods see [28]).

### Data processing

**Figure 2:**
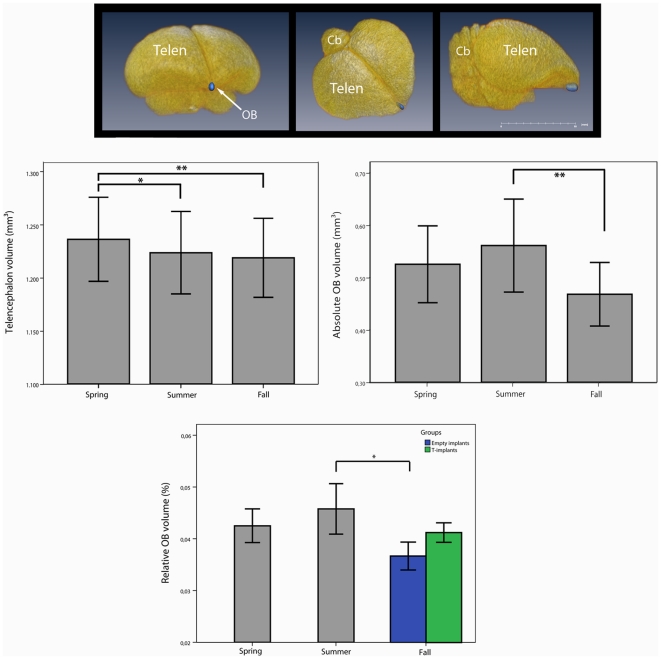
Telencephalon and olfactory bulb volumes. **Top**: 3D surface rendering of the starling brain. The olfactory bulb (OB) is indicated in blue. Notice the small size of the OB compared to the rest of the brain. Cb  =  Cerebellum; Telen  =  Telencephalon. **Middle**: Telencephalon volumes (left) and absolute OB volumes (right) of the same individuals (N = 14) at different times of the year. (*p<0.05; **p<0.001). Error bars represent standard deviation. **Bottom**: Because of the significant seasonal changes in telencephalon volume, the OB volumes are expressed relative to the volume of the telencephalon (in %). Relative OB volumes of the same individuals (N = 14) at different times of the year. After the experiment in summer the starlings were split up into two groups, one received empty implants (N = 9, blue), the other testosterone filled implants (N = 5; green). Error bars represent standard deviation. (*p<0.05).

Based on the 2D IR MRI images, the calculation of quantitative T_1_-maps in which the grey level of each pixel represents the fit-parameter T_1_ was carried out using in-house software developed in Matlab (The Mathworks, USA). The volume of the olfactory bulb was delineated for each subject on the T_1_-weighted 3D using Amira software (Visage Imaging, Berlin, Germany), whereby volumes could be calculated from the voxel size (0.098×0.098×0.50 mm^3^) and the number of voxels of the ROI (**Fig. 2**). All data calculations/delineations were done blind, by giving each acquired data set a random code.

### Statistical analyses

#### T_1_ measures

T_1_ gives us a direct indication of how much manganese has been incorporated in to the OB (shorter T_1_ =  more manganese). First we used a linear mixed model (LMM) analysis, with a first order autoregressive (AR1) model of the covariance between stimuli and between groups (T- implants vs. empty implants) for the T_1_ measures to see if testosterone had an effect on the OB activity during the non-breeding season (fall). The LMM analysis included as fixed effects one between-subject factor (group) with two levels, and one within-subject factor (stimuli) with two levels based on the two stimuli (milfoil vs. no scent) and bird ID as random effect. If the group x stimuli interaction effect was significant, then the group factor (T-implants vs. empty implants) would be included into the overall LMM over the different seasons (see below) as a between-subject factor with two levels, otherwise it would be left out. LMM analysis was then done with a first order autoregressive (AR1) model of the covariance between stimuli and seasons (spring, summer and fall) for the T_1_ measures. The LMM analysis included as fixed effects two within-subject factors (stimuli and season) with stimuli having each two levels (milfoil vs. no scent) and season having three levels (spring, summer and fall) and bird ID as random effect. If the stimuli x season interaction effect was statistically significant, then *post hoc* simple main effects were examined that compared stimuli differences for each season using the Bonferroni correction for multiple comparisons.

#### Discriminatory ability

Discriminatory ability (DA = [100×(T1 milfoil - T1 no scent)/T1 no scent)]) was calculated per season. To see if testosterone had an effect on the OB activity during the non-breeding season (fall), an LMM analysis with a first order autoregressive (AR1) model of the covariance between groups (T- implants vs. empty implants) for the DA measures was done. If the group effect was significant, then the group factor (T-implants vs. empty implants) would be included into the overall LMM over the different seasons (see below) as a between-subject factor with two levels, otherwise it would be left out. LMM analysis was then done with a first order autoregressive (AR1) model of the covariance between seasons (spring, summer and fall) for the DA measures. The LMM analysis included as fixed effect one within-subject factor (season) with three levels (spring, summer and fall) and bird ID as random effect. If the season effect was statistically significant, then *post hoc* simple main effects were examined that compared seasonal differences using the Bonferroni correction for multiple comparisons.

#### Volumes (telencephalon, absolute and relative OB)

To see if testosterone had an effect during the non-breeding season (fall), an LMM analysis with a first order autoregressive (AR1) model of the covariance between groups (T- implants vs. empty implants) for the volume measures was done. If the group effect was significant, then the group factor (T-implants vs. empty implants) would be included into the overall LMM over the different seasons (see below) as a between-subject factor with two levels, otherwise it would be left out. LMM analysis was then done with a first order autoregressive (AR1) model of the covariance between stimuli and seasons (spring, summer and fall) for the T_1_ measures. The LMM analysis included as fixed effect one within-subject factor (season) with three levels (spring, summer and fall) and bird ID as random effect. If the season effect was statistically significant, then *post hoc* simple main effects were examined that compared seasonal differences using the Bonferroni correction for multiple comparisons. Differences were considered significant for an alpha level of *P*<0.05. All data were analysed using SPSS 16.0 (SPSS inc.).

## Results

### Seasonal differences in OB activity and volumes

There was a statistically significant interaction effect of season x stimuli for T1 (*F* = 4.68; df = 2, 53.86; *P* = 0.013). *Post hoc* comparisons for the three seasons (spring, summer and fall) found a significant stimuli difference (milfoil vs. no scent) only for spring (*F* = 5.35; df = 1, 10.55; *P* = 0.042), with milfoil stimulation showing a higher T_1_ in the OB (**Fig. 1**).

There was a statistically significant seasonal effect for DA (*F* = 11.1; df = 2, 27.51; *P*<0.001), telencephalon volume (*F* = 7.44; df = 2, 24.96; *P* = 0.003), and absolute OB volume (*F* = 9.42; df = 2, 25.18; *P* = 0.001). *Post hoc* pair-wise comparisons of DA for the three seasons (spring, summer and fall) found that it was significantly higher for spring (4.78±3.9%) compared to summer (−0.59±1.9%; *P* = 0.006) and compared to fall (0.02±3.3%; *P* = 0.001) (**Fig. 1**). *Post hoc* pair-wise comparisons of telencephalon volume for the three seasons (spring, summer and fall) found that it was significantly larger for spring (1238±41 mm^3^) compared to summer (1228±40 mm^3^; *P* = 0.028) and compared to fall (1220±38 mm^3^; *P* = 0.002) (**Fig. 2**), also a trend was observed between the volume of summer compared to that of fall (*P* = 0.063). *Post hoc* pair-wise comparisons of absolute OB volume for the three seasons (spring, summer and fall) found that it was significantly larger for summer (0.56±0.09 mm^3^) compared to fall (0.47±0.06 mm^3^; *P* = 0.001) (**Fig. 2**) and a trend was observed between the volume of spring (0.53±0.07 mm^3^) compared to that of fall (*P* = 0.079).

### The effect of testosterone on the olfactory bulb (OB) during the non-breeding season

Beaks of all T-implanted males were entirely yellow indicating elevated levels of testosterone and those of the empty-implanted males were black indicative of basal levels of testosterone. In agreement with this, circulating plasma T levels of the T-implanted group were significantly higher (mean: 3.66±1.90 SE ng/ml) than the empty implanted group (mean: 1.46±1.39 SE ng/ml; t-test: *t*_11_ = −2.4, *p* = 0.037). T-levels of 3.66 ng/ml are in the natural range: in an aviary group of male starlings mean T-levels around 3 ng/ml plasma were measured [23]. Despite these physiological differences between the two groups no group difference could be found regarding T_1_ measures (*F* = 0.26; df = 1, 12.26; *P* = 0.644) nor a group x stimulus effect (*F* = 1.55; df = 1, 10.9; *P* = 0.239).

Also no group differences could be found in discriminatory ability (DA) (*F* = 1.43; df = 1, 10; *P* = 0.260), telencephalon volume (*F* = 0.60; df = 1, 12; *P* = 0.455) or absolute OB volume (*F* = 2.37; df = 1, 12; *P* = 0.150). There was however a trend for relative OB volume (*F* = 3.93; df = 1, 12; *P* = 0.071) with the T-implanted group having a larger relative OB volume (0.041±0.003%) compared to the control group (0.037±0.004%) (**Fig. 2**).

Seeing as the relative OB volume showed a trend between the T-implanted and the control group, a LMM analysis with season as within-factor was done for each group separately. For the control group a statistically significant seasonal effect (*F* = 4.74; df = 2, 15.77; *P* = 0.024) was found, no such seasonal effect was found for the T-implanted group (*F* = 3.05; df = 2, 8.28; *P* = 0.102). *Post hoc* pair-wise comparisons of relative OB volume for the control group for the three seasons (spring, summer and fall) found that the relative OB volume was significantly larger for summer (0.043±0.005%) compared to fall (0.037±0.004%; *P* = 0.025) (**Fig. 2**).

## Discussion

We have demonstrated by means of Manganese Enhanced MRI that the olfactory bulb (OB) of the starling exhibits seasonal plasticity to a specific plant odour. We have shown (I) that the OB is only able to readily detect milfoil odour during the breeding season, (II) that there is less manganese in the OB when milfoil odour is offered, (III) that the OB size changes over the year (even within non-breeding context) and (IV) that exogenous testosterone implants have an effect on the (relative) OB size during the non-breeding season.

A clear difference in activity of the OB was seen between the breeding and non-breeding season. In spring (breeding season) the OB of starlings showed a significantly different uptake of manganese when an odour (in this case biologically relevant milfoil) was presented than without any specific odour (background odours). This different response to the milfoil odour was not observed during the subsequent non-breeding season measurements. The starling's olfactory responsiveness thus appears to coincide with the reproductive season. These results confirm a previous report, in which the starling's ability to respond to conditioned odour cues was only present during the reproductive period [8]. The authors suggested that the olfactory sensitivity is linked with photoperiodically controlled endocrine production and reproductive behaviours. Our study tested for the first time if plasma T has an effect on the olfactory sensitivity, by artificially enhancing the normally basal plasma T levels during the non-reproductive period to levels typical for the reproductive period. After 3 weeks of high plasma T levels no statistical difference in the olfactory DA could be observed between T-implanted and empty implanted birds. Of course a statistical non-effect is very hard to interpret and caution should be taken when assigning any biological relevance to this observation. It should be noted however that this is only the first step in unravelling the mechanisms behind this seasonal change in olfactory acuity. T was the most obvious target to start investigating seeing the role it plays in seasonal changes of the song control system in songbirds [9], but other non-steroidal factors could of course also play specific roles in the seasonal change in olfactory acuity like photoperiod, melatonin levels, presence of females, etc.

Although the OB's ability to distinguish an odour changed with reproductive context, the volume of the OB followed a different time-course over the year. It would seem that the OB size remained somewhat constant (enlarged) in spring (breeding season) and summer (non-breeding season) but only becomes smaller towards the end of the year (fall). This indicates that anatomical structure and function are not always linked. This is in agreement with a previous study which highlighted the lack of correlation between discriminatory ability (or acuity) and relative OB size in 5 species of passerines [29] but in contrast to findings from the passerine song control nuclei, where seasonal changes in the volume are linked to differences in the song output [9]. In humans a similar lack of correlation between olfactory discrimination and OB size has recently been shown [30]. It also illustrates that for European starlings summer is a somewhat special case of the non-breeding season. Had starlings been measured only in spring and fall, we would have incorrectly concluded that structure and function of the OB are linked (large DA and large OB in spring vs. small DA and small OB in fall). We also showed that T-implants do have an effect on OB size, it would seem that T-implanted birds have an enlarged OB compared to the empty-implanted starlings. The size of the OB of empty-implanted birds is smaller in fall compared to the rest of the year, however for the T-implanted birds this is not the case, the relative OB size remains the same compared to the rest of the year.

We have shown recently that the telencephalon of starlings changes significantly from spring to summer and that this somewhat small change can only be detected when using repeated measurements in the same individuals [31]. In this study we also show small but significant differences of the telencephalon over the year. The effect of T on OB size could not have been seen if OB size was not expressed relative to the telencephalon volume indicating the importance of relative sizes especially in species with very plastic brains such as songbirds.

We expected that the amount of manganese accumulation would be higher in the olfactory bulb when the birds perceived a biologically relevant odour like milfoil. To our surprise we found the opposite. During the breeding season the amount of manganese accumulation (after 1 hour of Mn^2+^ injection) with milfoil stimulus was significantly lower than without an odour. Because the manganese was only administered one hour before the measurements into the nostrils it would be highly unlikely that more manganese would have left the OB and reached the telencephalon. Manganese can be seen at the OB of starlings at about 40 min after its introduction in the nostrils, and it is cleared from the OB 150 min thereafter. This timing occurs regardless if milfoil is used as stimulus (data not shown). Because both stimuli showed the same clearance ratio we would have to conclude that not more manganese has left the OB but that less manganese has reached the OB when milfoil is used as stimulus. The organization of olfactory pathways in vertebrates and invertebrates shares a number of common features [32]. Presynaptic inhibition of olfactory afferents has been shown to be a common functional strategy in mice [33], turtle [34], and even insects [35]. Presynaptic inhibition is regulated through calcium channel inhibition [33], [34]. Because Manganese uptake into the neurons is mediated by the same calcium channels [24], the presynaptic inhibition of olfactory receptor neurons would reduce the manganese uptake by these neurons and could explain the lower manganese accumulation we found in the OB of starlings stimulated with an odour.

The underlying anatomical or physiological mechanisms responsible for the seasonal change in olfactory acuity are however not explained. A possible hypothesis could be that starlings show a seasonal change in the amount of olfactory receptors in the nasal epithelium, as found in red-backed salamanders (*Plethodon cinereus*)[36]. A reduced number of olfactory receptors could lead to an overall decrease in olfactory acuity during the non-breeding season, possibly complemented, by the previously mentioned pre-synaptic inhibition altering between seasons. In rats dopaminergic modulation appears to play a role in altering odour discrimination [37] and dopamine has been shown to regulate other seasonally changing regions and behaviour in European starlings [38].

In summary, we demonstrate here seasonal changes in the ability of the starlings' OB to readily detect an odour emanated under natural conditions from a plant preferred as green nest material by male starling. The results of this experiment add to our knowledge of the proximate mechanisms underlying olfactory perception in adult vertebrates in three important ways. First, the results of the seasonal experiment provide a demonstration of dissociation of OB volume changes over the year and OB activity over the year in starlings. The size of the olfactory bulb is not related to its functional activity over the different seasons. Second, the results suggest that starling's olfactory system distinguishes odours on the basis of presynaptic inhibition and that this mechanism alters between seasons. Third, the results of the testosterone implantation experiment suggest that testosterone level is not directly responsible (at least not on its own) for changes in olfactory bulb activity in male starlings but has an effect on olfactory bulb size. These results shed insight into the plasticity of the olfactory system as governed by the reproductive cycle. Further investigations using MRI in correlation with anatomical and electrophysiological approaches are necessary to further explore the possible relationship between seasonal changes in steroids, photoperiod and OB activity.

## References

[1] Roper TJ (1999). Olfaction in birds.. Advances in the Study of Behaviour.

[2] Balthazart J, Taziaux M (2009). The underestimated role of olfaction in avian reproduction?. Behav Brain Res.

[3] Hagelin JC, Jones IL (2007). Bird odors and other chemical substances: A defense mechanism or overlooked mode of intraspecific communication?. AUK.

[4] Hagelin JC, Jamieson BGM (2006). Odors and chemical signalling.. Reproductive Behavior and Phylogeny of Aves.

[5] Gwinner H (1997). The function of green plants in nests of European starlings (Sturnus vulgaris).. Behaviour.

[6] Clark L, Mason JR (1985). Use of nest material as insecticidal and anti-pathogenic agents by the European starling.. Oecologia.

[7] Gwinner H, Oltrogge M, Trost L, Nienaber U (2000). Green plants in starling nests: effects on nestlings.. Anim Behav.

[8] Clark L, Smeraski CA (1990). Seasonal shifts in odor acuity by starlings.. J Exp Zool.

[9] Ball GF, Auger CJ, Bernard DJ, Charlier TD, Sartor JJ (2004). Seasonal plasticity in the song control system: multiple brain sites of steroid hormone action and the importance of variation in song behavior.. Ann N Y Acad Sci.

[10] Riters LV, Eens M, Pinxten R, Duffy DL, Balthazart J (2000). Seasonal changes in courtship song and the medial preoptic area in male European starlings (Sturnus vulgaris).. Horm Behav.

[11] Shepherd GM (2004). The human sense of smell: are we better than we think?. PLoS Biol.

[12] Meredith M (1991). Sensory processing in the main and accessory olfactory systems: comparisons and contrasts.. J Steroid Biochem Mol Biol.

[13] Tirindelli R, Dibattista M, Pifferi S, Menini A (2009). From Pheromones to Behavior.. Physiological Reviews.

[14] Steiger SS, Fidler AE, Valcu M, Kempenaers B (2008). Avian olfactory receptor gene repertoires: evidence for a well-developed sense of smell in birds?. Proc Biol Sci.

[15] Glusman G, Yanai I, Rubin I, Lancet D (2001). The complete human olfactory subgenome.. Genome Res.

[16] Pautler RG, Silva AC, Koretsky AP (1998). In vivo neuronal tract tracing using manganese-enhanced magnetic resonance imaging.. Magn Reson Med.

[17] Pautler RG, Koretsky AP (2002). Tracing odor-induced activation in the olfactory bulbs of mice using manganese-enhanced magnetic resonance imaging.. Neuroimage.

[18] Koretsky AP, Silva AC (2004). Manganese-enhanced magnetic resonance imaging (MEMRI).. NMR Biomed.

[19] Chuang KH, Lee JH, Silva AC, Belluscio L, Koretsky AP (2009). Manganese enhanced MRI reveals functional circuitry in response to odorant stimuli.. Neuroimage.

[20] Van Meir V, Verhoye M, Absil P, Eens M, Balthazart J (2004). Differential effects of testosterone on neuronal populations and their connections in a sensorimotor brain nucleus controlling song production in songbirds: a manganese enhanced-magnetic resonance imaging study.. Neuroimage.

[21] Van Meir V, Pavlova D, Verhoye M, Pinxten R, Balthazart J (2006). In vivo MR imaging of the seasonal volumetric and functional plasticity of song control nuclei in relation to song output in a female songbird.. NeuroImage.

[22] Tindemans I, Boumans T, Verhoye M, Van der Linden A (2006). IR-SE and IR-MEMRI allow in vivo visualization of oscine neuroarchitecture including the main forebrain regions of the song control system.. NMR Biomed.

[23] Gwinner H, Gwinner E, Dittami J (1987). Effects of nestboxes on LH, testosterone, testicular size and reproductive behavior of male European starlings in spring.. Behavior.

[24] Narita K, Kawasaki F, Kita H (1990). Mn and Mg influxes through Ca channels of motor nerve terminals are prevented by verapamil in frogs.. Brain Res.

[25] Dawson A (1983). Plasma gonadal steroid levels in wild starlings (Sturnus vulgaris) during the annual cycle and in relation to the stages of breeding.. Gen Comp Endocrinol.

[26] Ball GF, Wingfield JC (1987). Changes in plasma-levels of luteinizing-hormone and sex steroid-hormone in relation to multiple-broodedness and nest-site density in male starlings.. Physiological Zoology.

[27] De Ridder E, Pinxten R, Mees V, Eens M (2002). Short- and long-term effects of male-like concentrations of testosterone on female European starlings (Sturnus vulgaris).. AUK.

[28] Roelants H, Schneider F, Goritz F, Streich J, Blottner S (2002). Seasonal changes of spermatogonial proliferation in roe deer, demonstrated by flow cytometric analysis of c-kit receptor, in relation to follicle-stimulating hormone, luteinizing hormone, and testosterone.. Biol Reprod.

[29] Clark L, Avilova KV, Bean NJ (1993). Odor Thresholds in Passerines.. Comparative Biochemistry and Physiology A-Physiology.

[30] Haehner A, Rodewald A, Gerber JC, Hummel T (2008). Correlation of olfactory function with changes in the volume of the human olfactory bulb.. Arch Otolaryngol Head Neck Surg.

[31] De Groof G, Verhoye M, Poirier C, Leemans A, Eens M (2009). Structural changes between seasons in the songbird auditory forebrain.. J Neurosci.

[32] Hildebrand JG, Shepherd GM (1997). Mechanisms of olfactory discrimination: converging evidence for common principles across phyla.. Annu Rev Neurosci.

[33] Wachowiak M, McGann JP, Heyward PM, Shao Z, Puche AC (2005). Inhibition [corrected] of olfactory receptor neuron input to olfactory bulb glomeruli mediated by suppression of presynaptic calcium influx.. J Neurophysiol.

[34] Wachowiak M, Cohen LB (1999). Presynaptic inhibition of primary olfactory afferents mediated by different mechanisms in lobster and turtle.. J Neurosci.

[35] Distler PG, Boeckh J (1997). Synaptic connections between identified neuron types in the antennal lobe glomeruli of the cockroach, Periplaneta americana: II. Local multiglomerular interneurons.. J Comp Neurol.

[36] Dawley EM, Nelsen M, Lopata A, Schwartz J, Bierly A (2006). Cell birth and survival following seasonal periods of cell proliferation in the chemosensory epithelia of red-backed salamanders, Plethodon cinereus.. Brain Behav Evol.

[37] Yue EL, Cleland TA, Pavlis M, Linster C (2004). Opposing effects of D1 and D2 receptor activation on odor discrimination learning.. Behav Neurosci.

[38] Heimovics SA, Riters LV (2008). Evidence that dopamine within motivation and song control brain regions regulates birdsong context-dependently.. Physiol Behav.

